# *AP1S3* Mutations Cause Skin Autoinflammation by Disrupting Keratinocyte Autophagy and Up-Regulating IL-36 Production

**DOI:** 10.1016/j.jid.2016.06.618

**Published:** 2016-11

**Authors:** Satveer K. Mahil, Sophie Twelves, Katalin Farkas, Niovi Setta-Kaffetzi, A. David Burden, Joanna E. Gach, Alan D. Irvine, László Képíró, Maja Mockenhaupt, Hazel H. Oon, Jason Pinner, Annamari Ranki, Marieke M.B. Seyger, Pere Soler-Palacin, Eoin R. Storan, Eugene S. Tan, Laurence Valeyrie-Allanore, Helen S. Young, Richard C. Trembath, Siew-Eng Choon, Marta Szell, Zsuzsanna Bata-Csorgo, Catherine H. Smith, Paola Di Meglio, Jonathan N. Barker, Francesca Capon

**Affiliations:** 1 Division of Genetics and Molecular Medicine, King’s College London, London, UK; 2 MTA-SZTE Dermatological Research Group, Szeged, Hungary; 3 Department of Dermatology, University of Glasgow, Glasgow, UK; 4 Department of Dermatology, Birmingham Children’s Hospital, Birmingham, UK; 5 Paediatric Dermatology, Our Lady’s Children’s Hospital, Dublin, Ireland; 6 Department of Dermatology and Allergology, University of Szeged, Hungary; 7 Dokumentationszentrum schwerer Hautreaktionen (dZh) and RegiSCAR-study, Department of Dermatology, Medical Center–University of Freiburg, Freiburg, Germany; 8 National Skin Centre, Singapore; 9 Department of Medical Genomics, Royal Prince Alfred Hospital, Camperdown, Australia; 10 Department of Skin and Allergic Diseases, Helsinki University Central Hospital, Helsinki, Finland; 11 Department of Dermatology, Radboud University Nijmegen Medical Centre, Nijmegen, The Netherlands; 12 Pediatric Infectious Diseases and Immunodeficiencies Unit, Hospital Universitari Vall d'Hebron, Barcelona, Spain; 13 Department of Dermatology, University Hospital, Galway, Ireland; 14 Department of Dermatology, Henri Mondor Hospital, Paris, France; 15 Department of Dermatology, University of Manchester; 16 Department of Dermatology, Hospital Sultanah Aminah, Johor Bahru, Malaysia; 17 Institute of Medical Genetics, University of Szeged, Hungary; 18 Mill Hill Laboratory, The Francis Crick Institute, London, UK

**Keywords:** 3-MA, 3-methyladenine, AID, autoinflammatory disorder, CRISPR, clustered regularly-interspaced short palindromic repeats, Cas9, CRISPR-associated endonuclease 9, GFP, green fluorescent protein, MALP-2, macrophage-activating lipopeptide 2, siRNA, small interfering RNA, TLR-2/6, Toll-like receptor 2/6

## Abstract

Prominent skin involvement is a defining characteristic of autoinflammatory disorders caused by abnormal IL-1 signaling. However, the pathways and cell types that drive cutaneous autoinflammatory features remain poorly understood. We sought to address this issue by investigating the pathogenesis of pustular psoriasis, a model of autoinflammatory disorders with predominant cutaneous manifestations. We specifically characterized the impact of mutations affecting *AP1S3*, a disease gene previously identified by our group and validated here in a newly ascertained patient resource. We first showed that *AP1S3* expression is distinctively elevated in keratinocytes. Because *AP1S3* encodes a protein implicated in autophagosome formation, we next investigated the effects of gene silencing on this pathway. We found that *AP1S3* knockout disrupts keratinocyte autophagy, causing abnormal accumulation of p62, an adaptor protein mediating NF-κB activation. We showed that as a consequence, *AP1S3*-deficient cells up-regulate IL-1 signaling and overexpress IL-36α, a cytokine that is emerging as an important mediator of skin inflammation. These abnormal immune profiles were recapitulated by pharmacological inhibition of autophagy and verified in patient keratinocytes, where they were reversed by IL-36 blockade. These findings show that keratinocytes play a key role in skin autoinflammation and identify autophagy modulation of IL-36 signaling as a therapeutic target.

## Introduction

Autoinflammatory disorders (AIDs) are a group of inherited conditions caused by abnormal activation of the innate immune system. AIDs typically present with recurrent and seemingly unprovoked episodes of systemic upset, which are almost invariably accompanied by joint and skin inflammation (Aksentijevich and Kastner, 2011). The latter can manifest with urticarial, pustular, or ulcerative eruptions, which are considered important markers of disease activity (Beer et al., 2014).

In the last 15 years, genetic studies have identified more than 30 AID genes, illuminating fundamental innate immune pathways and highlighting pathogenic mechanisms (most notably, abnormal IL-1 production) that have been successfully targeted by therapeutic interventions (de Jesus et al., 2015).

Despite these successes, the basis of organ-specific disease manifestations is still unclear. This is particularly true of skin pathology, because the nature of the cells and molecular mechanisms that mediate cutaneous inflammation in AID remain poorly defined (Beer et al., 2014).

We sought to address this issue by investigating the pathogenesis of pustular psoriasis, a severe AID manifesting with repeated eruptions of painful skin pustules. These can be localized to the palms and soles (palmar plantar pustulosis), toes and fingertips (acrodermatitis continua of Hallopeau) or affect most of the body surface (generalized pustular psoriasis). Although the lesions can be accompanied by arthritis and systemic upset, cutaneous involvement is the most prominent clinical feature of the disease (Griffiths and Barker, 2010). This makes pustular psoriasis an ideal model for investigating the molecular mechanisms that drive skin inflammation in AID.

**Figure 1:**
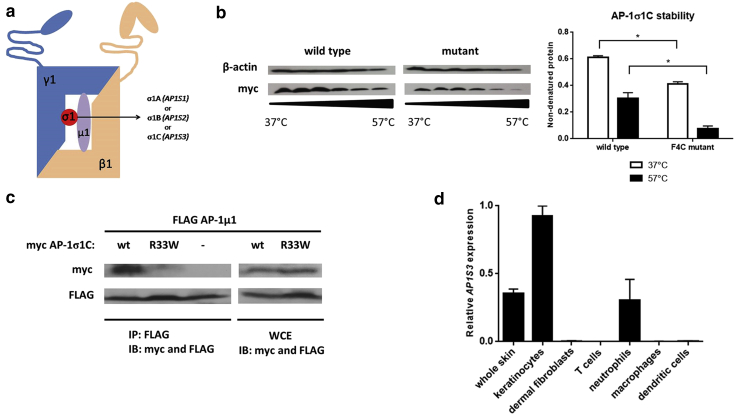
***AP1S3* loss-of-function mutations are most likely to affect skin keratinocytes.** (**a**) Schematic representation of AP-1 structure. (**b**) HEK293 cells were transfected with wild-type and mutant *AP1S3* constructs. Lysates were subjected to the indicated temperature gradient, and soluble (nondenatured) proteins were analyzed by Western blotting. The densitometry shows the fraction of nondenatured protein (mean ± standard error of the mean of the results obtained in two experiments). (**c**) HEK293 cells were transfected with myc-tagged *AP1S3* and FLAG-tagged *AP1M1* constructs. Lysates were subjected to immune precipitation (IP) and immune blotting (IB) as indicated. The image is representative of results obtained in two experiments. (**d**) Real-time PCR analysis showing abundant *AP1S3* expression in keratinocytes. The data show the mean ± standard error of the mean of measurements obtained in two donors. ^∗^*P* ≤ 0.05. IB, immune blotting; IP, immune precipitation; WCE, whole cell extracts; wt, wild type.

We specifically investigated the pathogenic role of *AP1S3*, a gene that we found to be mutated in all forms of pustular psoriasis (Setta-Kaffetzi et al., 2014). *AP1S3* encodes a subunit of AP-1, a heterotetramer that mediates membrane trafficking between the post-Golgi network and the endosome (Robinson, 2004). The complex is composed of two large (AP-1γ1 and AP-1β1), one medium (AP-1μ1) and one small subunit (AP-1σ1). The latter exists in three alternative forms (AP-1σ1A, AP-1σ1B and AP-1σ1C), encoded by paralogous genes (*AP1S1*, *AP1S2*, *AP1S3*), so that the *AP1S3* product is AP-1σ1C (Figure 1a). The σ1 subunit confers stability to AP-1 tetramers, so that mutations in *AP1S* genes are expected to disrupt the entire complex (Robinson, 2004).

The AP-1 complex has also been implicated in the formation of autophagosomes (Guo et al., 2012). These are specialized vesicles that mediate the degradation of cellular components by autophagy, a catabolic process that can be activated by nutrient stress (e.g., starvation). Given that autophagy modulates cytokine production downstream of various pattern recognition receptors (Netea-Maier et al., 2016), we hypothesized that *AP1S3* mutations would disturb autophagic activity, causing innate immune dysregulation. We then validated our pathogenic model in a variety of in vitro experimental systems and in patient cells.

## Results

### Validation of *AP1S3* as a pustular psoriasis gene

Although we previously reported that two *AP1S3* mutations (p.Phe4Cys and p.Arg33Trp) account for approximately 10% of European pustular psoriasis patients (Setta-Kaffetzi et al., 2014), the rarity of the disease has hindered the replication of this finding. To address this issue, we screened the *AP1S3* coding region in 85 newly ascertained patients (53 European and 32 non-European subjects) (see Supplementary Table S1 online), recruited across Europe and East Asia. This uncovered p.Phe4Cys and p.Arg33Trp alleles in five unrelated individuals (n = 3 generalized pustular psoriasis and n = 2 palmar plantar pustulosis patients) (Table 1). All were of European descent, confirming the limited geographic distribution of the two mutations. Two of the three generalized pustular psoriasis patients carried the *AP1S3* mutation in conjunction with a deleterious change in *IL36RN*, a pustular psoriasis gene encoding the IL-36 receptor antagonist (Marrakchi et al., 2011, Onoufriadis et al., 2011). One of these individuals exhibited a particularly severe, recalcitrant phenotype and had a sister with a milder form of the disease, who only carried the *IL36RN* mutation (see Supplementary Table S2 online).

Taken together, these observations validate the involvement of *AP1S3* in pustular psoriasis and suggest the possibility of epistasis between *IL36RN* and *AP1S3* alleles.

### *AP1S3* mutations disrupt protein function in keratinocytes

Structural homology modeling indicates that the p.Phe4Cys change maps to a β-sheet required for protein folding, whereas the p.Arg33Trp substitution is expected to disrupt the interaction between AP-1σ1C and AP-1μ1A (Setta-Kaffetzi et al., 2014). This strongly suggests that both mutations are loss-of-function alleles.

To validate these predictions, we first examined the effect of p.Phe4Cys on the thermal stability of AP-1σ1C. After transfection of wild-type and mutant *AP1S3* constructs into HEK293 cultures, we subjected cell lysates to a temperature gradient and monitored AP-1σ1C levels by western blotting. We found that p.Phe4Cys proteins were denatured significantly more quickly than their wild-type counterparts (Figure 1b), confirming that the mutation disrupts AP-1σ1C stability.

To investigate the impact of the p.Arg33Trp allele, we carried out co-immunoprecipitation experiments, using FLAG-*AP1M1* and myc-*AP1S3* constructs transfected into HEK293 cells. As expected, we found that wild-type myc-AP1σ1C co-precipitated with FLAG-AP1μ1A. This interaction, however, was disrupted when FLAG-*AP1M1* was co-transfected with a p.Arg33Trp myc-*AP1S3* cDNA (Figure 1c). Similar results were obtained in immunofluorescence experiments, showing that wild-type myc-AP1σ1C co-localized with FLAG-AP1μ1A, whereas the mutant p.Arg33Trp protein did not (see Supplementary Figure S1 online). Thus, we concluded that the p.Arg33Trp mutation disturbs the interaction between AP-1σ1C and AP-1μ1A, as predicted in-silico.

Having validated the loss-of-function nature of disease alleles, we sought to establish which cell types are most likely to be affected by *AP1S3* deficiency. We therefore measured gene expression in biologically relevant cell populations. Although transcript levels were low in neutrophils and virtually undetectable in CD4^+^ T lymphocytes, we observed abundant gene expression in keratinocytes (Figure 1d). The impact of disease alleles was therefore modeled in this cell type.

### *AP1S3* deficiency causes impaired keratinocyte autophagy

Because autophagosome formation requires a functional AP-1 complex (Guo et al., 2012), we hypothesized that *AP1S3* loss-of-function mutations may disrupt keratinocyte autophagy.

**Figure 2:**
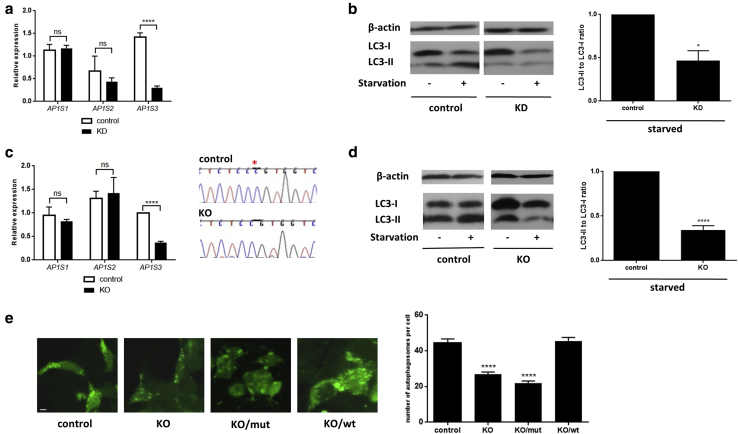
***AP1S3* deficiency results in impaired autophagy.** (**a**) After the generation of a HaCaT *AP1S3* knockdown cell line, gene silencing was measured by real-time PCR, because of cross-reactivity of the anti-AP1σ1c antibody with the proteins encoded by *AP1S1* and *AP1S2*. (**b**) Starvation-induced LC3-II accumulation was measured by Western blotting and densitometry. The data are presented as mean ± standard error of the mean of measurements obtained in four independent experiments. (**c**) HEK293 *AP1S3* knockout cell lines harboring a c.124delC change (highlighted by a red asterisk in the chromatogram) were generated by CRISPR/Cas-9 editing. (**d**) Cells were starved to induce autophagy, and LC3-II accumulation was measured by Western blotting. The data are presented as described. (**e**) Control and *AP1S3* KO HEK293 cells were transfected with GFP-LC3 and either an empty vector (control and KO panels) or a rescue construct (wild-type *AP1S3* in KO/wt panel and p.Arg33Trp *AP1S3* in KO/mut). Starvation-induced LC3 punctae were visualized by confocal fluorescence microscopy. The data are presented as mean ± standard error of the mean of measurements obtained in at least 15 cells per experiment. Scale bar = 5 μm. ^∗^*P* ≤ 0.05, ^∗∗∗∗^*P* ≤ 0.0001. Cas9, CRISPR-associated protein-9; CRISPR, clustered regularly interspaced short palindromic repeats; GFP, green fluorescent protein; KD, knockdown; KO, knockout; mut, mutated; ns, not significant; wt, wild-type.

We first examined this possibility in a HaCaT keratinocyte cell line stably transduced with a silencing *AP1S3* small hairpin RNA (Setta-Kaffetzi et al., 2014) (Figure 2a). After inducing autophagy by starvation, we monitored the conversion of the LC3-I protein into its modified form (LC3-II), which is a well-recognized autophagosome marker (Klionsky et al., 2012). We found that LC3-II levels were significantly reduced in *AP1S3* knockdown versus control cell lines (Figure 2b).

We then repeated the experiment in a HEK293 *AP1S3* knockout cell line, generated by clustered regularly interspaced short palindromic repeats (CRISPR)–CRISPR-associated endonuclease-9 (Cas9) genome editing (Figure 2c). This confirmed that *AP1S3* silencing causes a very significant decrease in starvation-induced LC3-II accumulation (Figure 2d).

To further validate our findings, we used fluorescence microscopy to visualize the expression of LC3-green fluorescent protein (GFP) constructs transfected into the HEK293 *AP1S3* knockout cell line. We found that the number of autophagosomes that had incorporated LC3-GFP was significantly reduced in knockout versus control cells. This phenotype was rescued by the overexpression of wild-type but not mutant (p.Arg33Trp) *AP1S3* constructs (Figure 2e).

Thus, *AP1S3* deficiency disrupts autophagy induction in multiple experimental systems.

### *AP1S3* deficiency results in abnormal p62 accumulation and enhanced Toll-like receptor (TLR) 2/6 signaling

**Figure 3:**
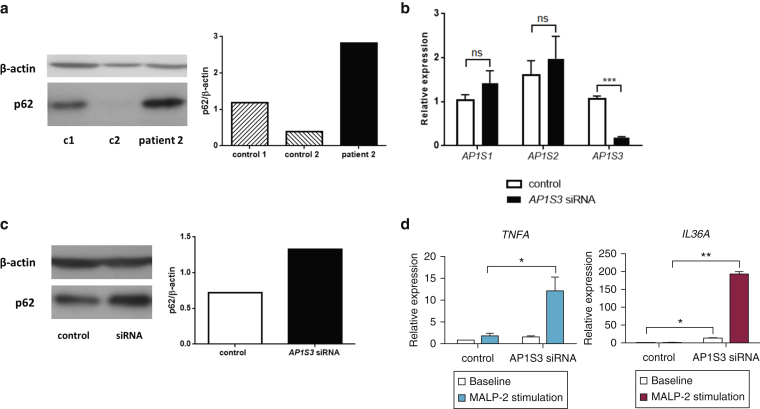
**Abnormal p62 accumulation and enhanced TLR-2/6 signaling in *AP1S3*-deficient keratinocytes.** (**a**) p62 levels were measured in patient and control subject keratinocytes by Western blotting and densitometry. (**b**) After the transfection of silencing (*AP1S3* siRNA) and nonsilencing (control) siRNA pools into primary keratinocytes, (**c**) baseline p62 levels were measured by Western blotting and densitometry. (**d**) Alternatively, cells were stimulated with MALP-2 in triplicate, and the induction of TLR2/6-dependent genes was measured by real-time PCR. The data are representative of results obtained in at least two independent experiments and are presented as mean ± standard error of the mean of duplicate stimulations. ^∗^*P* ≤ 0.05, ^∗∗^*P* ≤ 0.01, ^∗∗∗^*P* ≤ 0.001. c, control; ns, not significant; siRNA, small interfering RNA; TLR, Toll-like receptor.

It has been shown that keratinocyte autophagy modulates NF-κB activation downstream of TLR-2/6 by regulating the degradation of the p62 adaptor protein (Lee et al., 2011). This led us to hypothesize that *AP1S3* deficiency would cause an abnormal accumulation of p62, resulting in enhanced NF-κB signaling. We therefore measured p62 protein levels in keratinocytes cultured from the hair plucks of one affected individual (carrying the *AP1S3* p.Arg33Trp mutation) and two healthy control subjects. We found that p62 expression was markedly increased in the patient’s cells (Figure 3a). A similar up-regulation was observed in normal primary keratinocytes transfected with *AP1S3* small interfering RNAs (siRNAs) (Figure 3b and 3c) and in a HaCaT *AP1S3* knockout cell line (see Supplementary Figure S2a and b online). We therefore concluded that the abnormal p62 accumulation observed in the patient was a result of *AP1S3* deficiency.

To further explore these findings, we measured macrophage-activating lipopeptide 2 (MALP-2)–induced cytokine expression in primary keratinocytes transiently transfected with *AP1S3* siRNAs (Figure 3d). Although there was no *IL1B*, *IL6,* or *IL8* induction at the examined time point, we detected a marked increase in *TNFA* levels. We also observed a significant induction of *IL36A* (but not *IL36B* or *IL36G*), a cytokine that drives abnormal immune signaling in patients with *IL36RN* mutations (Onoufriadis et al., 2011). Importantly, the induction of *TNFA* and *IL36A* was significantly enhanced in *AP1S3*-deficient cells compared with control (Figure 3d).

We then repeated the MALP-2 stimulations in the HaCaT *AP1S3* knockout cell line. This confirmed the abnormal induction of *TNFA* and *IL36A* in knockout cells (see Supplementary Figure S2c).

### *AP1S3* deficiency causes abnormal IL-1 signaling and up-regulates baseline IL-36 expression

**Figure 4:**
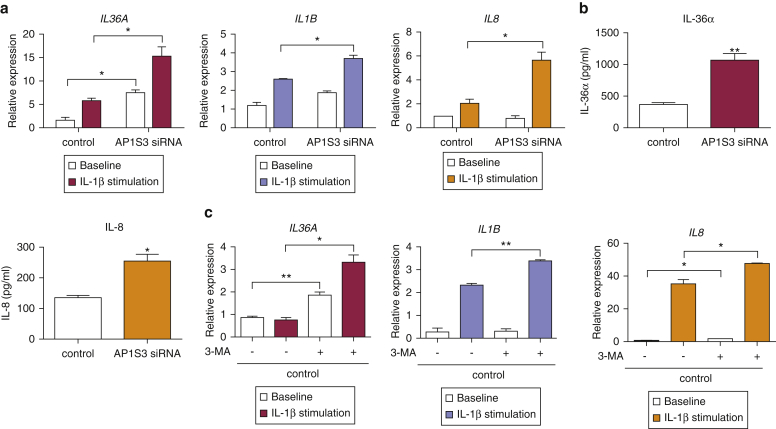
***AP1S3*-deficient primary keratinocytes exhibit an abnormal immune profile, which can be recapitulated by autophagy inhibition.** (**a**) After siRNA-mediated *AP1S3* silencing, primary keratinocytes were stimulated with IL-1β, and gene expression was determined by real-time PCR. (**b**) Alternatively, cells were cultured for a further 48 hours in the absence of stimuli, and cytokine production was measured by ELISA. (**c**) Normal primary keratinocytes were cultured in the presence or absence of 3-MA and subsequently stimulated with IL-1β. Gene expression was determined by real-time PCR. All data are representative of results obtained in two independent experiments and are presented as mean ± standard error of the mean of (**a**) duplicate or (**b, c**) triplicate measurements. ^∗^*P* ≤ 0.05, ^∗∗^*P* ≤ 0.01. 3-MA, 3-methyladenine; siRNA, small interfering RNA.

Autophagy-mediated degradation of p62 also regulates IL-1 signaling (Lee et al., 2012), a response that plays a major role in autoinflammation. To determine whether *AP1S3* deficiency would also affect this pathway, we transfected primary keratinocytes with *AP1S3* siRNA pools and measured cytokine levels after IL-1 stimulation. Although *TNFA* expression was unchanged at the examined time point, we observed a clear up-regulation of *IL1B*, *IL8,* and *IL36A* transcripts. The induction of all cytokines was markedly up-regulated in *AP1S3*-deficient cells compared with control (Figure 4a). These observations were replicated in HaCaT *AP1S3*-knockout cells (see Supplementary Figure S3a online), thus validating the effects of gene silencing on IL-1 signaling.

Surprisingly, our experiments showed that baseline *IL36A* expression was markedly increased in *AP1S3-*deficient cells, both at the RNA and protein levels (Figures 4a and b, and see Supplementary Figure S2c). A similar, although less pronounced, effect was also observed for *IL36B* and *IL36G* mRNA expression (see Supplementary Figures S4a and S4b online) and IL-8 protein secretion (Figure 4b).

To determine whether this up-regulation was also a consequence of impaired autophagy, we cultured normal primary keratinocytes in medium supplemented with 3-methyladenine (3-MA), an agent that blocks the formation of autophagosomes (Klionsky et al., 2012). As expected, we found that 3-MA treatment caused an increase in IL-1–dependent cytokine expression. *IL36A* baseline expression was also up-regulated by 3-MA (Figure 4c). These observations, which were replicated in HaCaT keratinocytes (see Supplementary Figure S3b), show that the proinflammatory effects of *AP1S3* deficiency are mediated by a disruption of keratinocyte autophagy.

### Patients harboring *AP1S3* mutations up-regulate IL-36 expression and IL-1 signaling

**Figure 5:**
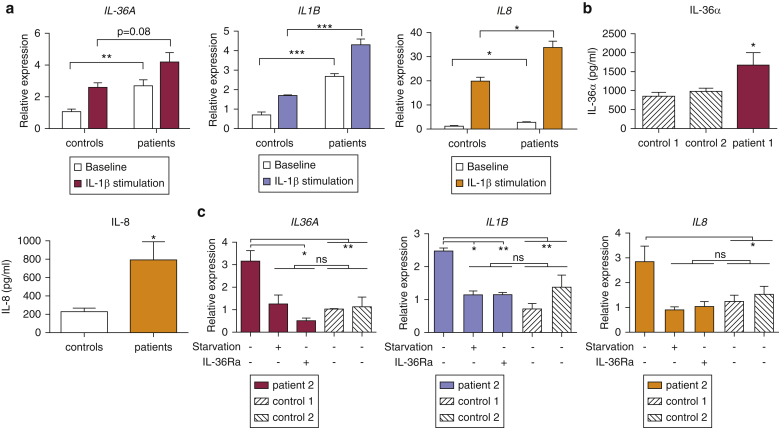
**Abnormal cytokine expression in the keratinocytes of patients harboring *AP1S3* mutations.** (**a**) Primary keratinocytes were stimulated with IL-1β, and cytokine induction was measured by real-time PCR. The data are presented as mean ± standard error of the mean of duplicate stimulations carried out in the cells of two unrelated patients and two healthy control subjects. (**b**) IL-36α and IL-8 production was measured in culture supernatants by ELISA. Data are presented as mean ± standard error of the mean of triplicate measurements. (**c**) Primary keratinocytes were starved to induce autophagy or cultured in the presence of IL-36Ra. Gene expression was measured by real-time PCR. The data are presented as mean ± standard error of the mean of triplicate measurements, obtained in one patient and two healthy control subjects. ^∗^*P* < 0.05, ^∗∗^*P* < 0.01, ^∗∗∗^*P* < 0.001. ns, not significant.

To validate the pathophysiological relevance of our findings, we cultured keratinocytes from the hair plucks of two affected individuals (each carrying an *AP1S3* mutation and a wild-type *IL36RN* sequence) and two healthy control subjects. Although we observed only a weak response to MALP-2 stimulation, we found that cytokine levels were robustly up-regulated after IL-1 treatment. Importantly, the induction of *IL1B, IL8,* and *IL36A* transcripts was increased in the keratinocytes of patients compared with control (Figure 5a), replicating the results generated in *AP1S3*-knockdown cells.

The basal expression of IL-36 cytokines was also up-regulated in patient keratinocytes (Figure 5a and b and see Supplementary Figure S4c), further validating the data obtained in *AP1S3*-deficient cells. *IL1B* and *IL8* baseline transcripts were also significantly overexpressed in the examined individuals (Figure 5a).

To further investigate the mechanisms underlying these observations, we measured transcript levels after autophagy induction by starvation, or blockade, of the IL-36 receptor with a recombinant antagonist (IL-36Ra). We found that both treatments could lower patient cytokine expression to the levels observed in healthy control subjects (Figure 5c, and see Supplementary Figure S4d). Although the experiment was carried out in a single patient, the results were also replicated in *AP1S3*-knockout cells (see Supplementary Figure S5 online), suggesting that impaired autophagy and enhanced IL-36 signaling both contribute to the abnormal immune profile associated with *AP1S3* mutations.

## Discussion

The aim of our study was to characterize the molecular mechanisms underlying the cutaneous features of AIDs. We therefore investigated the pathogenesis of pustular psoriasis, focusing our attention on *AP1S3,* a gene that is specifically mutated in this disease. We first validated the pathogenic involvement of this locus by demonstrating the presence of disease alleles in five of the 53 European patients (9.4%) who were included in our screening. We observed that *AP1S3* mutations can be inherited in conjunction with *IL36RN* changes, modifying the phenotypic effect of the latter. This suggests that *AP1S3* alleles may exacerbate the effects of *IL36RN* deficiency by disturbing IL-36 homeostasis, a notion that is borne out by the results of our functional studies.

First, our experiments showed that *AP1S3* expression was low or undetectable in cells that do not respond to IL-36 stimulation (neutrophils and CD4^+^ T cells), whereas transcript levels were abundant in keratinocytes, where IL-36 signaling can be activated by TLR agonists (Gabay and Towne, 2015). The only other known gene for pustular psoriasis (*CARD14*) is also abundantly expressed in keratinocytes (Berki et al., 2015), suggesting that these cells play an important role in cutaneous autoinflammation. This is in keeping with the emerging view of keratinocytes as immune sentinels contributing to host defense through the engagement of innate receptors and the production of proinflammatory mediators (Di Meglio et al., 2011, Lowes et al., 2013).

The involvement of *AP1S3* in IL-36 regulation is also supported by repeated observations of increased *IL36A* expression in *AP1S3*-deficient cells and in nonlesional keratinocytes, derived from patient hair plucks. Of note, stable *AP1S3* knockout also led to constitutive up-regulation of *IL1B* and *IL8* (see Supplementary Figure S3). Although this phenotype mirrored the expression profile observed in patients, it was not replicated in the transient gene-silencing experiments, where mRNA levels were measured shortly after knockdown initiation. Although *IL36A* was up-regulated at this early time point, the other two cytokines were not, suggesting that the overexpression of *IL1B* and *IL8* may be secondary to IL-36 accumulation. Indeed, our experiments showed that IL-36 receptor blockade is sufficient to normalize *IL1B* and *IL8* levels in patient keratinocytes.

Thus, our observations place IL-36 at the center of a proinflammatory loop that drives enhanced cytokine production in skin autoinflammation (see Supplementary Figure S6 online). This is in keeping with the results of recent studies, indicating that *IL36A* is markedly up-regulated in psoriatic skin and that this is unlikely to be a secondary consequence of inflammation (Swindell et al., 2016). Given that therapeutics blocking IL-36 are now under development (Wolf and Ferris, 2014), these discoveries have important translational implications.

Our experiments show that the effects of *AP1S3* mutations on cytokine production are mediated by disruption of keratinocyte autophagy, causing abnormal p62 accumulation and enhanced NF-κB activation downstream of TLR-2/6 and IL-1R. Of note, p62 transcripts are up-regulated in psoriatic lesions, whereas the expression of molecules that contribute to skin inflammation is reduced in p62-deficient keratinocytes (Lee et al., 2011).

Here, IL-1 treatment of patient cells (which overexpress p62) caused a moderate (∼2-fold) induction of *IL1B* transcripts (Figure 5) but a substantial up-regulation of *IL8* (>20-fold). Given that the latter cytokine plays a fundamental role in driving neutrophilic skin infiltration, this finding has a clear pathological relevance.

Autophagy can also modulate cytokine production at the posttranslational level, by degrading components of the inflammasome, the molecular complex that cleaves pro-IL1β into a bioactive molecule (Shi et al., 2012). Although this process has been chiefly investigated in mouse macrophages, it might also be active in human keratinocytes, where it could amplify the effects of *AP1S3* mutations.

It is now widely accepted that perturbations of protein degradation play a pathogenic role in various AIDs with prominent dermatological features (Martinon and Aksentijevich, 2015). Evidence recently generated in animal models also indicates that therapeutic effects of anakinra (an IL-1 blocker widely used for the treatment of AIDs) are partly mediated by the rescue of defective autophagy (Iannitti et al., 2016). In the light of this evidence, our work warrants further studies of impaired keratinocyte autophagy as a pathogenic mechanism and therapeutic target in skin autoinflammation.

## Methods

### Participants

This study was performed in accordance with the declaration of Helsinki and was approved by the ethics committees of participating institutions. Written informed consent was obtained from all participants. Patients were ascertained by trained dermatologists (see Supplementary Table S3 online) on the basis of established diagnostic criteria (Griffiths and Barker, 2010). Patients 1 and 2 were described elsewhere as T002206 and T001882, respectively (Setta-Kaffetzi et al., 2014). Healthy volunteers were recruited within King’s College London. All affected individuals were screened for *IL36RN* and *AP1S3* mutations as described (Onoufriadis et al., 2011, Setta-Kaffetzi et al., 2014).

### Plasmids and constructs

The wild-type and mutant myc-tagged *AP1S3* constructs are described elsewhere (Setta-Kaffetzi et al., 2014). The FLAG-*AP1M1* construct was generated by cloning the gene coding sequence into a c-Flag pcDNA3 vector (Addgene #20011). CRISPR/Cas9 guide RNAs (see Supplementary Table S4 online) were designed with the CRISPR design tool (http://crispr.mit.edu/) and cloned into a pSpCas9BB-2A-GFP vector (Addgene #48138), as described elsewhere (Ran et al., 2013). All constructs were validated by Sanger sequencing.

### Primary cell culture

Primary keratinocytes and dermal fibroblasts were isolated from healthy skin discarded after plastic surgery. The keratinocytes were maintained in Epilife keratinocyte medium supplemented with Supplement 7 and 1% penicillin-streptomycin, and the fibroblasts were grown in DMEM supplemented with 10% fetal bovine serum and 1% penicillin-streptomycin (all reagents from Gibco, Waltham, MA).

Keratinocytes were derived from hair plucks as described elsewhere (Aasen and Izpisua Belmonte, 2010). Briefly, 12 hairs were plucked from the temporal scalp and placed in mTeSR1 medium (Stem Cell Technologies, Vancouver, Canada) containing 1% penicillin-streptomycin and 250 ng/ml amphotericin B (Sigma, St. Louis, MO). Once outgrowths were visible, mTeSR1 was replaced with Epilife keratinocyte medium containing Supplement 7 and 1% penicillin-streptomycin. After 14 days, cells were stimulated.

### Thermal stability assay

HEK293 cells were transfected with the indicated constructs, using Lipofectamine 2000 (Life Technologies, Waltham, MA). Cell lysates were then incubated for 5 minutes across a 37–57 °C temperature gradient. Samples were centrifuged for 30 minutes at 13,000 rpm at 4 °C, and the soluble fraction (supernatant) was analyzed by Western blotting.

### CRISPR/Cas9 genome editing

The protocol described by Ran et al. (2013) was used to edit HaCaT and HEK293 cells maintained in complete DMEM. Briefly, the guide RNA construct was transfected into the cells, using Lipofectamine 2000. After 48 hours, GFP-positive cells were isolated by flow cytometry and seeded for clonal expansion. The resulting cell lines were validated by Sanger sequencing of the target region, paralogous loci, and off-target sites predicted by the CRISPR design tool. The expression of *AP1S1*, *AP1S2,* and *AP1S3* was also measured by real-time PCR. Control cells were transfected with an empty pSpCas9BB-2A-GFP vector.

### Cell stimulation

For autophagy induction, cells were starved for 18 hours in Hank’s Balanced Salt solution (Gibco), and protein extracts were analyzed by Western blotting. For autophagy inhibition, cells were pretreated with 10mmol/L of 3-MA (Sigma) for 5 hours and then stimulated with 20ng/ml of IL-1β (Sigma) for 2 hours, in the presence 3-MA.

Alternatively, primary or immortalized keratinocytes were treated with 100ng/ml of MALP2 (Bio-techne, Minneapolis, MN) for 42 hours, 20ng/ml of IL-1β for 2 hours, 100ng/ml of IL-36Ra (Bio-techne) for 5 hours or were starved as described.

For transient gene-silencing experiments, cells were transfected for 48 hours with 33 nmol/L of *AP1S3* ON-TARGET plus SMARTpool siRNA or ON-TARGETplus nontargeting siRNA (GE Dharmacon, Lafayette, CO) using Lipofectamine 2000 and stimulated as described above.

### Real-time PCR and ELISA

RNAs isolated from skin, lymphocytes, in vitro derived macrophages/dendritic cells, and neutrophils were provided by Frank Nestle, Susan John, Leonie Taams (King’s College London), and Benjamin Fairfax (Wellcome Trust Centre for Human Genetics, Oxford), respectively. All remaining RNAs were isolated using the RNeasy Mini Plus kit (Qiagen, Hilden, Germany). Gene expression was assessed by real-time PCR by using the primers listed in Supplementary Table S4 online. Transcript levels were normalized to *PPIA* or *B2M* expression, measured with Applied Biosystems (Foster City, CA) TaqMan probes. IL-36α and IL-8 production was measured with the Human IL36A ELISA Kit (Sigma) and Human IL-8 ELISA Kit (Sigma).

### Co-immunoprecipitation and Western blotting

A rabbit monoclonal anti-FLAG (1:50, Cell Signaling Technology, Danvers, MA) was used in co-immunoprecipitation experiments, whereas Western blots were probed with rabbit polyclonal anti-LC3 (Cell Signaling Technology), rabbit polyclonal anti-β actin (Cell Signaling Technology), rabbit polyclonal anti-p62 (Sigma), or mouse monoclonal anti-myc (Thermo Scientific, Waltham, MA) (all 1:1,000). Densitometry was undertaken with Image J software (Schneider et al., 2012).

### Immunofluorescence microscopy

In the co-localization experiments, HEK293 cells were co-transfected with the indicated constructs, using Lipofectamine 2000. After 24 hours, cells were fixed and incubated with 1:500 mouse monoclonal anti-myc (Cell Signaling Technology) and 1:600 rabbit monoclonal anti-FLAG. Slides were imaged by using a C2 confocal microscope (Nikon, Tokyo, Japan), and z-stack images of at least 15 cells per slide were taken.

In autophagy induction experiments, HEK293 cells were transfected with a pEGFP-LC3 plasmid (Addgene #24920) and the indicated construct, using Lipofectamine 2000. After 24 hours, cells were starved for 18 hours in Hank’s Balanced Salt solution supplemented with 0.1 μmol/L of Bafilomycin A1 (Sigma). Cells were imaged as described above and autophagosomes were counted by using NIS-Elements Advanced Research software (Nikon).

### Statistics

Means were compared with unpaired Student *t* tests. Error bars represent standard error of the mean.

## ORCID

Francesca Capon: http://orcid.org/0000-0003-2432-5793

Alan Irvine: http://orcid.org/0000-0002-9048-2044

## Conflict of Interest

Maja Mockenhaupt is the coordinator of the international RegiSCAR-project, which was/is funded (among others) by a consortium of pharmaceutical companies (Bayer Vital, Boehringer-lngelheim, Cephalon, GlaxoSmithKline, MSD Sharp and Dohme, Merck, Novartis, Pfizer, Roche, Sanofi-Aventis, Servier, Tibotec, Grünenthal, Falk Pharma, UCB Biopharma, AB-Science). Maja Mockenhaupt is also a member of expert panels/advisory boards in the field of severe cutaneous adverse reaction coordinated by pharmaceutical companies (Boehringer Ingelheim, Merck, Sanofi). She has also been an expert in litigations concerning Stevens Johnson syndrome/toxic epidermal necrolysis. Helen Young is/has been a consultant or speaker to Abbott/Abbvie, Amgen, Janssen-Cilag, Leo-Pharma, Novartis, Lily, Stiefel, Teva Pharmaceuticals, and Wyeth/Pfizer.

## Figures and Tables

**Table 1:** Clinical phenotype of affected individuals bearing *AP1S3* disease alleles

Patient ID	Sex	Ethnicity	Diagnosis	Concurrent PV	Age of Onset, years	*IL36RN* Genotype	*AP1S3* Genotype
T010091	F	European	GPP	U	68	p.Ser113Leu/–	p.Phe4Cys/–
T030865	F	European	GPP	N	<1	p.Ser113Leu/–	p.Phe4Cys/–
T016713	F	European	PPP	N	55	–/–	p.Arg33Trp/–
T026517	F	European	PPP	N	50	–/–	p.Arg33Trp/–
T028754	F	European	PPP	N	49	–/–	p.Arg33Trp/–

Abbreviations: F, female; GPP, generalized pustular psoriasis; ID, identifier; N, no; PPP, palmar plantar pustulosis; PV, psoriasis vulgaris; U, unknown.
