# Supplementary material for: AP1S3 Mutations Cause Skin Autoinflammation by Disrupting Keratinocyte Autophagy and Up-Regulating IL-36 Production
Source: J Invest Dermatol. 2016 Nov;136(11):2251–9. doi: 10.1016/j.jid.2016.06.618 (PMC5070969; doi:10.1016/j.jid.2016.06.618)
Supplement: Supplementary Figures S1–S6 and Supplementary Tables S1–S4 [file mmc1.pdf]

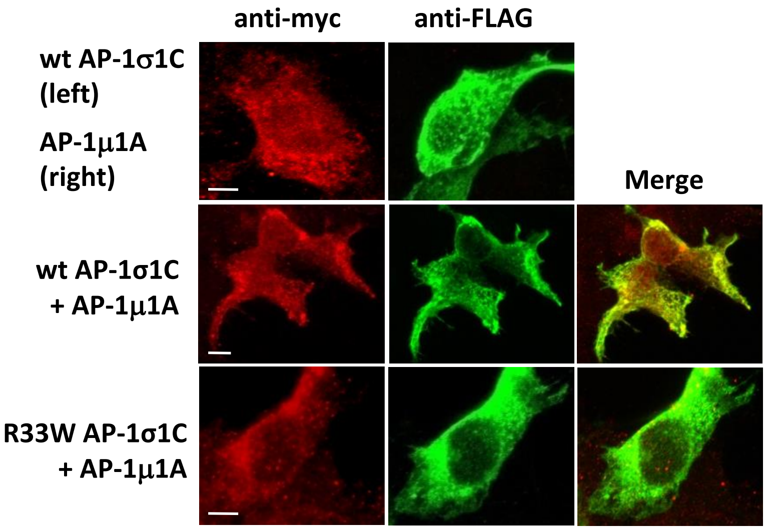

Figure S1. ***AP1S3*** **mutation disrupts the interaction between σ1C and μ1A subunits of AP-1.**
HEK293 cells were transfected with myc-tagged *AP1S3* and FLAG-tagged *AP1M1* constructs. The localisation of AP-1σ1C and AP-1μ1A proteins was visualised by confocal fluorescence microscopy. The images are representative of data obtained in ≥15 cells per experiment. Scale bar 5μm.

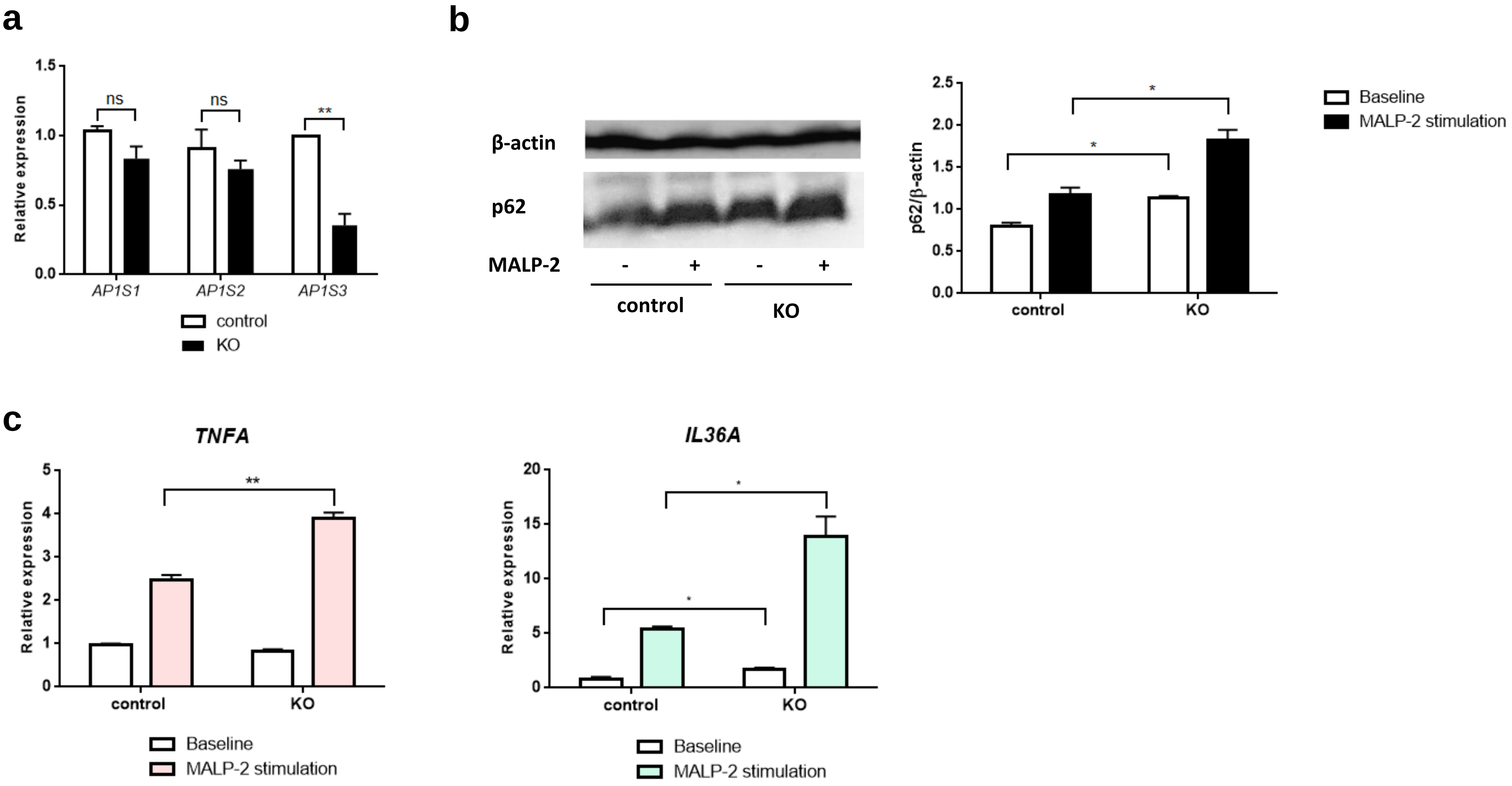

Figure S2. *AP1S3* deficient HaCaT keratinocytes exhibit abnormal p62 accumulation and enhanced TLR-2/6 signalling.
Following AP1S3 silencing by CRISPR/Cas9 genome editing (a), HaCaT keratinocytes were stimulated with MALP-2. p62 accumulation in control and knockout (KO) cells was monitored by western blotting and densitometry (b), and gene expression was determined by real-time PCR (c). The data are representative of results obtained in at least two independent experiments, and are presented as mean ± SEM of duplicate stimulations. $^{*}P \leq 0.05$, $^{**}P \leq 0.01$.

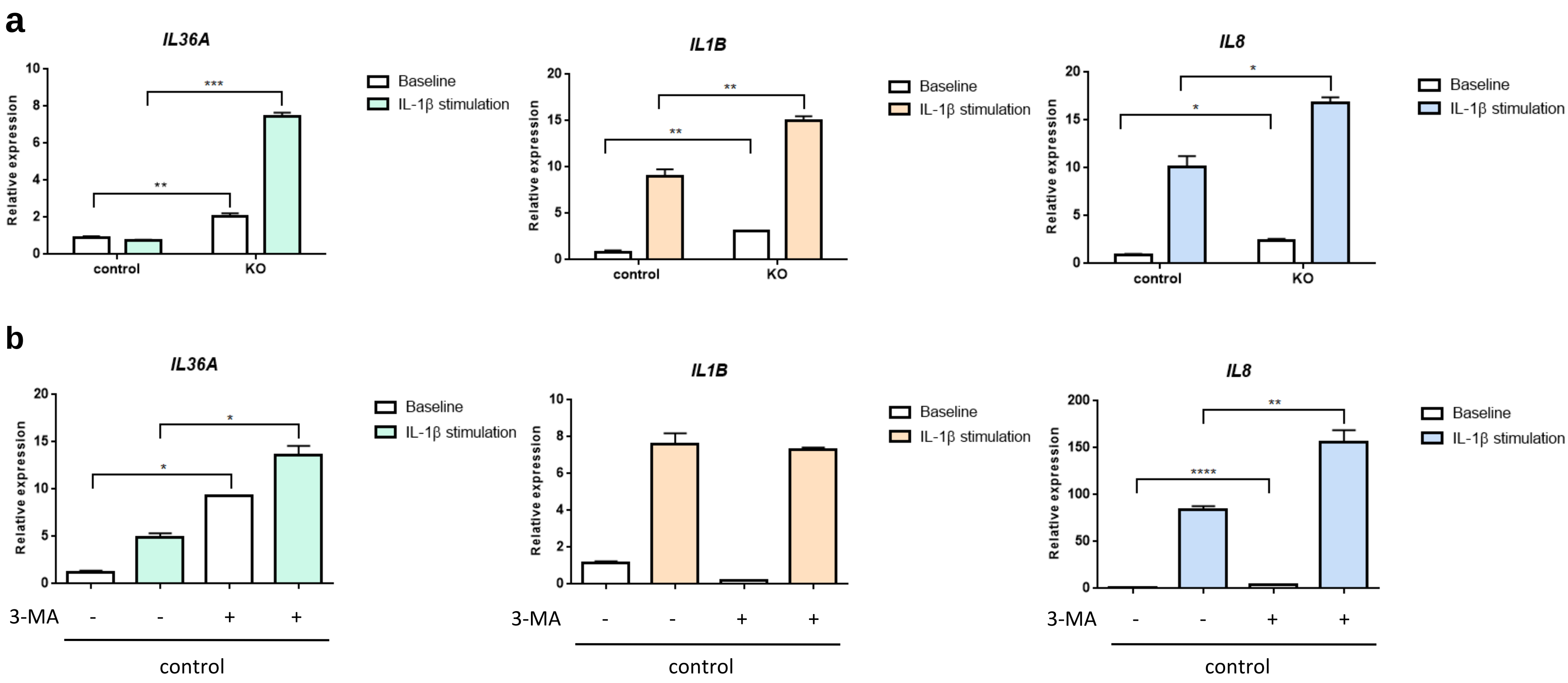

Figure S3. *AP1S3* deficient HaCaT keratinocytes exhibit an abnormal immune profile, which can be recapitulated by autophagy inhibition.

(a) Following *AP1S3* silencing by CRISPR/Cas-9 genome editing, control and *AP1S3* knockout (KO) cells were stimulated with IL-1β and gene expression was determined by real-time PCR. (b) Cells were cultured in the presence or absence of 3-MA and subsequently stimulated with IL-1β. Gene expression was determined by real-time PCR. All data are representative of results obtained in 2 independent experiments and are presented as mean ± SEM of triplicate measurements. $^{**}P \leq 0.01$, $^{***}P \leq 0.001$, $^{****}P \leq 0.0001$.

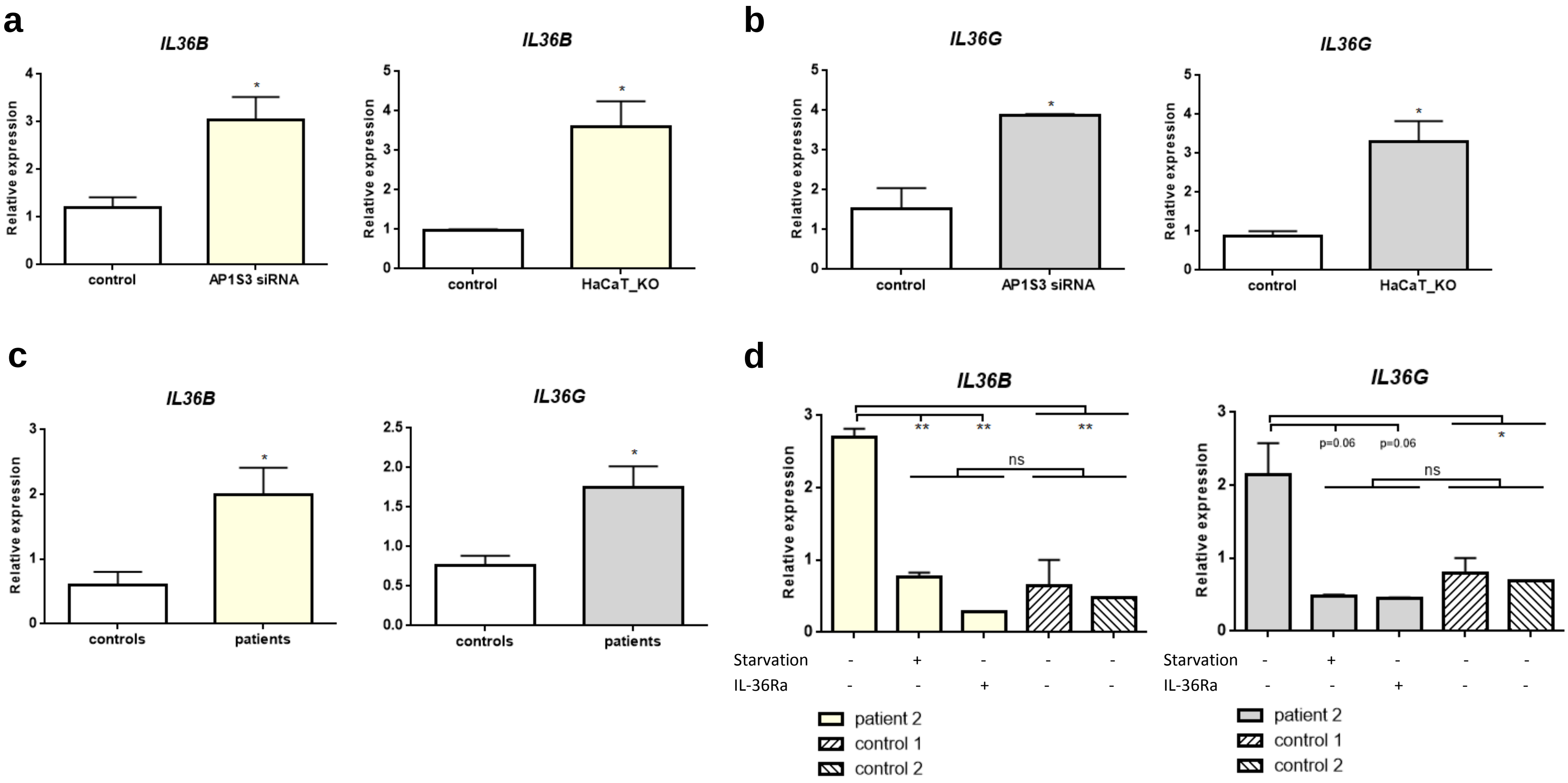

Figure S4. *IL36B* and *IL36G* expression is increased in *AP1S3* deficient cells. (a-b) Baseline gene expression was determined by real-time PCR, following *AP1S3* silencing by transient transfection of siRNA pools into primary keratinocytes (*AP1S3* siRNA) or CRISPR/Cas-9 genome editing of HaCaT keratinocytes (HaCaT_KO). Data are presented as mean ± SEM of triplicate measurements. (c) Baseline *IL36B* and *IL36G* expression was measured in primary keratinocytes from 2 unrelated patients harbouring *AP1S3* mutations and 2 healthy controls. The data are presented as mean ± SEM of duplicate measurements. (d) Primary keratinocytes were starved to induce autophagy or cultured in the presence of IL-36Ra. The data are presented as mean ± SEM of triplicate measurements, obtained in 1 patient and 2 healthy controls. KO, knockout. *P≤0.05, **P≤0.01.

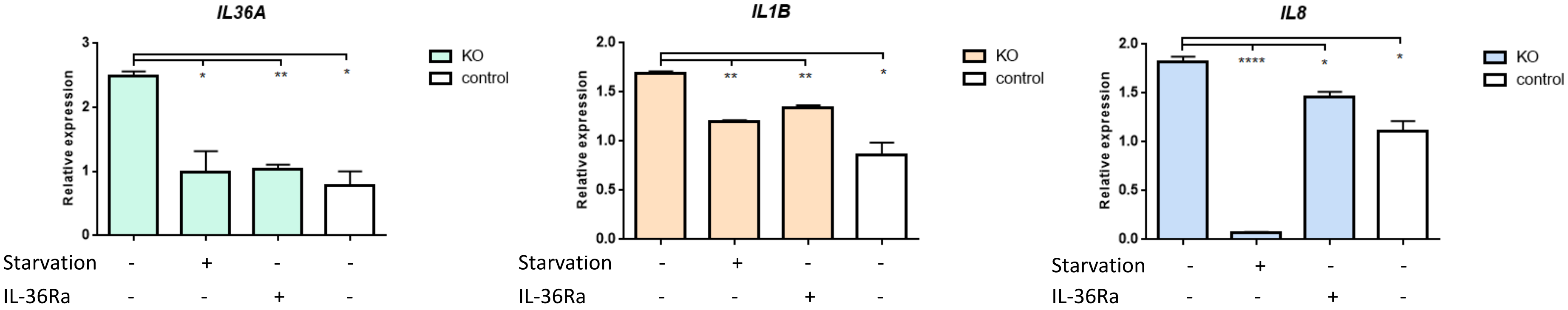

Figure S5. Autophagy induction and IL-36 blockade have anti-inflammatory effects on *AP1S3* deficient HaCaT keratinocytes.
Control and *AP1S3* knockout (KO) HaCaT keratinocytes were starved to induce autophagy or cultured in the presence of IL-36Ra. Gene expression was determined by real-time PCR. The data are representative of results obtained in two independent experiments and are presented as mean ± SEM of triplicate measurements. *$P \leq 0.05$, **$P \leq 0.01$, ****$P \leq 0.0001$.

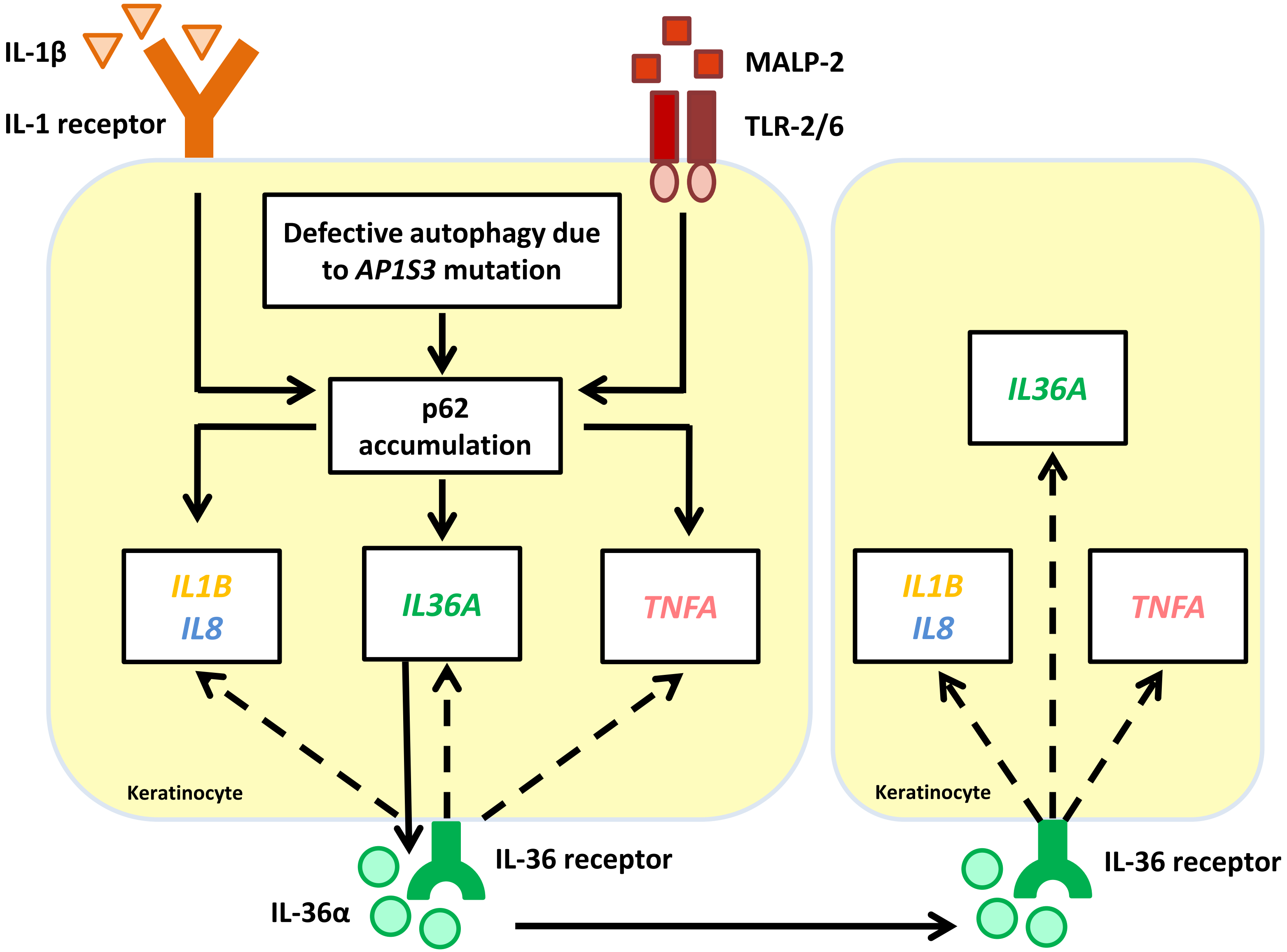

Figure S6. Proposed pathogenic model illustrating the pro-inflammatory effects of *AP1S3* deficiency.
AP1S3 mutations disrupt autophagy, causing abnormal accumulation of p62 and increased cytokine expression downstream of the IL-1 receptor and TLR-2/6. Among the cytokines that are up-regulated, IL-36 drives a positive feedback loop by inducing *IL36A*, *IL1B, IL8* and *TNF* expression.

Supplementary Table S1: Study cohort summary statistics

	**Clinical Diagnosis**			**Sex**		**Ethnicity**		
	**GPP**	**ACH**	**PPP**	**M**	**F**	**European**	**Asian**	**Other**
Patient	43	1	41	26	59	53	27	5
n.(%)	(50.6%)	(1.2%)	(48.2%)	(30.6%)	(69.4%)	(62.3%)	(31.8%)	(5.9%)

GPP: Generalised pustular psoriasis; ACH: Acrodermatitis continua of Hallopeau; PPP: Palmar plantar pustulosis. NB: all cases were pre-screened to exclude the presence of bi-allelic *IL36RN* mutations

Supplementary Table S2: Mutation status and disease features observed in two sisters with generalised pustular psoriasis

**Patient id**	**Sex**	***AP1S3* genotype**	***IL36RN* genotype**	**Age of onset**	**Disease course**	**PV concurrence**	**Systemic involvement**	**Drug response**
T030865	F	p.Phe4Cys/-	p.Ser113Leu/-	<1	Multiple flares	No	Yes	Partial response to infliximab
T030866	F	-/-	p.Ser113Leu/-	34	Single flare	No	Yes	Remission with methotrexate

Systemic involvement during flares was defined as the concurrence of fever>38°C and a neutrophil count >15*10^9/ml; PV: Psoriasis vulgaris

Supplementary Table S3: Details of patient recruitment centres

Patient ethnicity	Recruiting centre
European	European Registry of Severe Cutaneous Adverse Reactions
	Glasgow Western Infirmary, Glasgow, UK
	Helsinki University Central Hospital, Helsinki, Finland
	Our Lady's Children's Hospital, Dublin, Ireland
	Radboud University Nijmegen Medical Centre, Nijmegen, The Netherlands
	Royal Prince Alfred Hospital, Camperdown, Australia
	St John's Institute of Dermatology, London, UK
	University Hospital, Galway, Ireland
	University of Manchester, Manchester, UK
	University of Szeged, Szeged, Hungary
Asian	Birmingham Children's Hospital, Birmingham, UK
	Helsinki University Central Hospital, Helsinki, Finland
	Hospital SultanahAminah, Johor Bahru, Malaysia
	Hospital UniversitariValld'Hebron, Barcelona, Spain
	National Skin Centre, Singapore
Other	St John's Institute of Dermatology, London, UK

Supplementary Table S4: Oligonucleotide sequences used in the study

Target region	Primer ID	Sequence (5' to 3')	Annealing Temp (°C)	Application
AP1S3 Exon 1	AP1S3_ex1_Fwd	CTCCAGCGCTCCTTGCTC	56	Genome editing validation
	AP1S3_ex1_Rev	GGATCGAATGAATGAATGGA		
AP1S3 Exon 2	AP1S3_ex2_Fwd	TTTCAGTGTCTTTGCAGAACG	59	Genome editing validation
	AP1S3_ex2_Rev	CCCCAGCCTTCAAAGATTTC		
AP1S3 Exon 3	AP1S3_ex3_Fwd	GACTGCATATTCGTGGGAAAA	59	Genome editing validation
	AP1S3_ex3_Rev	GCTGAGATGGGGACTGTAGC		
AP1S3 Exon 4	AP1S3_ex4_Fwd	GGCAGATGTTTCCCCTGATA	59	Genome editing validation
	AP1S3_ex4_Rev	TCATCATCATCATCATCATCTTTC		
AP1S3 Exon 5	AP1S3_ex5_Fwd	ATTTCACAGTCTGCGGAAGG	59	Genome editing validation
	AP1S3_ex5_Rev	TGGGAGGCGTTGCTTATTTA		
AP1S2 Exon 2	AP1S2_ex2_Fwd	CATGTAAATGCCCCATCCCC	59	Sequencing of potential CRISPR off-target site
	AP1S2_ex2_Rev	TCACTGAAGTCTGCAATTTCCT		
AP1S1 Exon 2	AP1S1_ex2_Fwd	GGGCTGGATGTTGGAAGAAA	59	Sequencing of potential CRISPR off-target site
	AP1S1_ex2_Rev	GTCCTCCAGATGTCCTCAGC		
TREM1	TREM1_Fwd	CTGAAAAGCACAGGGTCAGG	56	Sequencing of potential CRISPR off-target site
	TREM1_Rev	ATGGATGTGGCTGGAAGTCA		
PALD1	PALD1_Fwd	TTTTGGTGAGGGTGCAAGC	59	Sequencing of potential CRISPR off-target site
	PALD1_Rev	CAGAAGGTGGAAAAGTGACCA		

FBXL5	FBXL5_Fwd	AGGACATTGTTGGACTAAGGACT	56	Sequencing of potential CRISPR off-target site
	FBXL5_Rev	TGAAGTAGGGCAGATCTTGGT		
TG	TG_Fwd	GCCTTCAACTCTGCCTTTATTCA	56	Sequencing of potential CRISPR off-target site
	TG_Rev	AAACCACAGAGCCAGCAGAA		
AP1S3	AP1S3_Fwd	GCTCTTCAGTCGACAAGGGA	60	Real-time PCR
	AP1S3_Rev	GCGTCAAGAGCTCATTGTCC		
AP1S2	AP1S2_Fwd	ACTGCAGGAGGAAGCTGAAAC	60	Real-time PCR
	AP1S2_Rev	GAAGCGGCTTAGCAAAACAGT		
AP1S1	AP1S1_Fwd	ATACTTTGGCAGTGTGTGCG	60	Real-time PCR
	AP1S1_Rev	CCTCTTGCAGTAGGTCAGCC		
TNF	TNF_Fwd	CCCAGGGACCTCTCTCTAATCA	60	Real-time PCR
	TNF_Rev	GCTACAGGCTTGTCACTCGG		
IL8	IL8_Fwd	TTGGCAGCCTTCCTGATTTC	60	Real-time PCR
	IL8_Rev	AACTTCTCCACAACCCTCT		
IL1B	IL1B_Fwd	GCCCTAAACAGATGAAGTGCTC	60	Real-time PCR
	IL1B_Rev	GAACCAGCATCTTCCTCAG		
IL36A	IL36A_Fwd	GGCCTGAATGGACTCAATCT	60	Real-time PCR
	IL36A_Rev	ACTTCACAGGCTCGGGTTG		
IL36B	IL36B_Fwd	CAGCATTAAGCCTGTCACTC	60	Real-time PCR
	IL36B_Rev	GCACAGAAGAGACAGAGATC		
IL36G	IL36G_Fwd	GGGCCGTCTATCAATCAATG	60	Real-time PCR
	IL36G_Rev	TGATAACAGCAACAGTGACTG		

AP1S3 exon 2	sgRNA-top	CACCGTTCAGATTATTCTCTCCCG	n/a	CRISPR guide RNA
AP1S3 exon 2	sgRNA-bottom	AAACCGGGAGAGAATAATCTGAAC	n/a	CRISPR guide RNA
Non-targeting	ON-TARGET plus Non-targeting pool siRNA	UGGUUUACAUGUCGACUAA<br>UGGUUUACAUGUUGUGUGA<br>UGGUUUACAUGUUUUCUGA<br>UGGUUUACAUGUUUUCCUA	n/a	Control oligonucleotides for siRNA mediated gene silencing
AP1S3 coding region	ON-TARGET plus SMARTpool Human *AP1S3*siRNA	CUACACAUGUUCUGGAUAA<br>AAGAAGAUCACCCGGGAAA<br>GGAAAUUACGGCUACAGAA<br>CAUUCAUAUUAGAGUGGUU	n/a	siRNA mediated gene silencing
AP1M1 coding region	AP1M1_HindIII_Fwd<br>AP1M1_BamHI_Rev	GTAAAGCTTATGTCCGCCAGCGCCGTCTAC<br>GATGGATCCCTGGGTCCGGAGCTGGTAATC	60	Cloning of *AP1M1* into c-Flag pcDNA3 plasmid
